# Phloretin, as a Potent Anticancer Compound: From Chemistry to Cellular Interactions

**DOI:** 10.3390/molecules27248819

**Published:** 2022-12-12

**Authors:** Hardeep Singh Tuli, Prangya Rath, Abhishek Chauhan, Seema Ramniwas, Kanupriya Vashishth, Mehmet Varol, Vivek Sheel Jaswal, Shafiul Haque, Katrin Sak

**Affiliations:** 1 Department of Biotechnology, Maharishi Markandeshwar Engineering College, Maharishi Markandeshwar (Deemed to be University), Mullana, Ambala 133207, India; 2 Amity Institute of Environmental Sciences, Amity University, Noida 201303, India; 3 Amity Institute of Environmental Toxicology, Safety and Management, Amity University, Noida 201303, India; 4 University Centre for Research & Development, University Institute of Pharmaceutical Sciences, Chandigarh University, Gharuan, Mohali 140413, India; 5 Advance Cardiac Centre Department of Cardiology, Post Graduate Institute of Medical Education and Research (PGIMER) Chandigarh, Chandigarh 160012, India; 6 Department of Molecular Biology and Genetics, Faculty of Science, Kotekli Campus, Mugla Sitki Kocman University, Mugla 48000, Turkey; 7 Department of Chemistry and Chemical Science, School of Physical & Material Sciences, Central University of Himachal Pradesh, Dharamshala 176206, India; 8 Research and Scientific Studies Unit, College of Nursing and Allied Health Sciences, Jazan University, Jazan 45142, Saudi Arabia; 9 NGO Praeventio, 50407 Tartu, Estonia

**Keywords:** phloretin, chemoprevention, apoptosis, anti-angiogenesis, anti-metastasis

## Abstract

Phloretin is a natural dihydrochalcone found in many fruits and vegetables, especially in apple tree leaves and the Manchurian apricots, exhibiting several therapeutic properties, such as antioxidant, antidiabetic, anti-inflammatory, and antitumor activities. In this review article, the diverse aspects of the anticancer potential of phloretin are addressed, presenting its antiproliferative, proapoptotic, antimetastatic, and antiangiogenic activities in many different preclinical cancer models. The fact that phloretin is a planar lipophilic polyphenol and, thus, a membrane-disrupting Pan-Assay Interference compound (PAIN) compromises the validity of the cell-based anticancer activities. Phloretin significantly reduces membrane dipole potential and, therefore, is expected to be able to activate a number of cellular signaling pathways in a non-specific way. In this way, the effects of this minor flavonoid on Bax and Bcl-2 proteins, caspases and MMPs, cytokines, and inflammatory enzymes are all analyzed in the current review. Moreover, besides the anticancer activities exerted by phloretin alone, its co-effects with conventional anticancer drugs are also under discussion. Therefore, this review presents a thorough overview of the preclinical anticancer potential of phloretin, allowing one to take the next steps in the development of novel drug candidates and move on to clinical trials.

## 1. Introduction

Despite advances in cell biology and medicinal chemistry, cancer has still been often considered an incurable disease [1]. Unfortunately, the incidence of this devastating disease is steadily increasing, outlined by a 47% rise in the global cancer burden expected from 2020 to 2040, implicating 28.4 million new cancer cases in 2040 [2]. Therefore, it is no surprise that the interest of the scientific community in natural anticancer agents has considerably increased during the last few decades [3]. Flavonoids are one of the most-studied plant secondary metabolites, revealing a diverse spectrum of different anticancer properties, such as anti-inflammatory, antiproliferative, cell-cycle arresting, proapoptotic, anti-invasive, antimetastatic, and antiangiogenic effects [4]. Based on their structural peculiarities, these polyphenolic phytochemicals are usually classified as flavanols or catechins, flavones, flavonols, flavanones, isoflavones, and anthocyanidins [5]. However, in addition to these major categories, the so-called minor types of flavonoids also exist, including aurones, chalcones, dihydrochalcones and dihydroflavonols, or flavononols [6]. Although a number of studies have demonstrated important anticancer activities of minor flavonoids, the current knowledge about their bioactivities in different malignant model systems is still much scarcer compared with the major types of flavonoids. Therefore, these less-studied agents might be even more attractive in studies aimed at identifying novel lead molecules for further efficient cancer therapeutics and describing new molecular targets for anticancer compounds.

Phloretin (dihydronaringenin or phloretol) is a dihydrochalcone belonging to the class of minor flavonoids. This natural polyphenol can be found in apple tree leaves and the Manchurian apricot [7]. Several studies have pointed to important anticancer properties of phloretin, describing its inhibitory action on the malignant progression in different preclinical models of lung cancer [8], oral cancer [9], esophageal cancer [7], gastric cancer [10,11], colon cancer [12,13], liver cancer [14], breast cancer [15,16], cervical cancer [17], and prostate cancer [18,19]. In doing so, phloretin can attack a number of molecular targets, modulating different intracellular signaling pathways [20]. In this review article, the cellular targets through which phloretin exerts its strong anticancer potential, behaving as an antiproliferative, proapoptotic, antiangiogenic, and antimetastatic compound, are highlighted. In addition, the co-effects between phloretin and traditional anticancer drugs currently used in clinical settings are also presented. Moreover, possible modes to improve the delivery of phloretin to cancer sites by using modern nanotechnological methods are under discussion. Hence, it is hoped that the current knowledge about the anticancer activities of phloretin, thoroughly presented in this review, will encourage further studies on this promising minor flavonoid, making a contribution to the complicated task of identifying novel efficient drugs in the fight against cancerous neoplasms.

## 2. Chemistry of the Molecule

**Figure 1:**
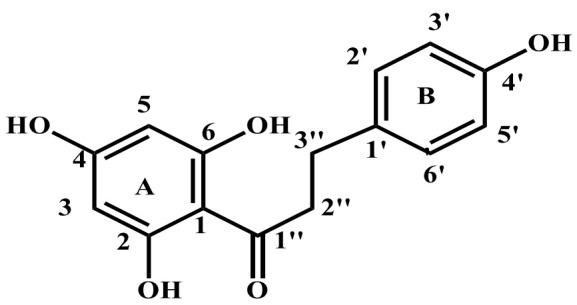
Chemical structure of phloretin.

**Figure 2:**
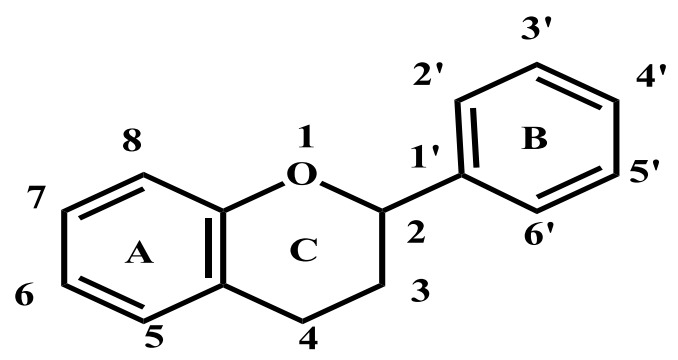
The basic structure of flavone.

The compound phloretin, i.e., 3-(4′-hydroxyphenyl)-1-(2,4,6-trihydroxyphenyl)propan-1-one, is a dihydrochalcone made up of two aromatic rings often referred to as A and B and containing hydroxy groups at positions 2, 4, 4′, and 6. (Figure 1) [21]. Phloretin has a wide range of unique biological functions due to its unique structure. Dihydrochalcones (also known as 1,3-diaryl-2-propen-1-ones) are phenolic compounds having a diphenylpropan (C6-C3-C6) flavonoid skeleton and no heterocyclic C ring. In fact, the majority of these secondary metabolites in plants are precursors of flavonoids (Figure 2) [22]. A wide range of pharmacological actions are produced by two aromatic phenol rings (rings A and B), hydroxyl groups, and a carbonyl group in phloretin.

**Figure 3:**
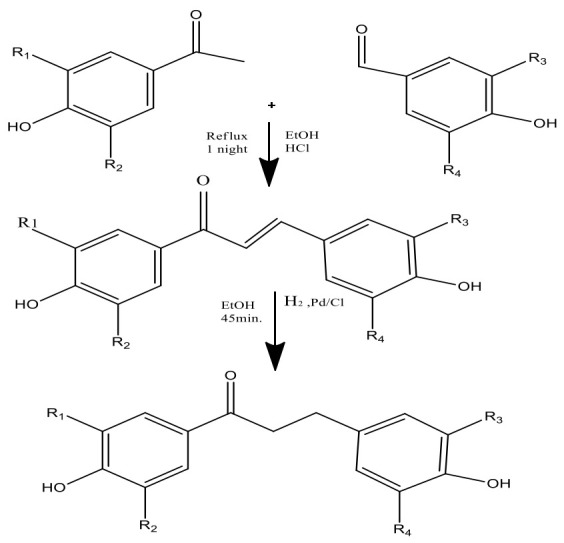
Two-step synthesis of phloretin (i.e., chalcones and dihydrochalcones).

The most common source of glycosylated phloretin derivatives is the apple tree (*Malus* spp.), which is a member of the Rosaceae family. *Malusdomestica* L. (apple) and *Fragaria × ananassa* Duchesne (strawberry), both members of the Rosaceae family, are widely used in the food industry and are excellent sources of flavonoids and phenolic compounds for the human diet [23]. Phloretin derivative concentrations can range significantly between apple fruit varieties and between different portions of the fruit. The apple tree’s leaves, barks, and roots have all yielded phloretin, which has also been separated from those sources [24]. The structure–activity relation obtained through the analysis of a broad range of flavonoids (Figure 3) revealed that the C2–C3 double bond and the neighboring di-OH at 3′ and 4′ in the B-ring are crucial for potent antiproliferative action [23]. The high antiproliferative activity requires both the C3-OH and C8-methoxyl groups in polymethoxylated flavones. For the efficient inhibition in each of those situations, di-OH3′, 4′, a double bond at C2–C3, and a carbonyl at C-4 were all necessary. It has been demonstrated that the di-OH 3′ and 4′ on the B-ring selectively affects Cdk2, causing G1 cell arrest [24]. The C2–C3 double bond, which affects the molecule’s planarity, and the presence and location of hydroxyl/*O*-methyl groups on the B-ring and A-ring, therefore, constitute the structural characteristics of flavonoids that have the greatest impact on cell proliferation [25].

**Figure 4:**
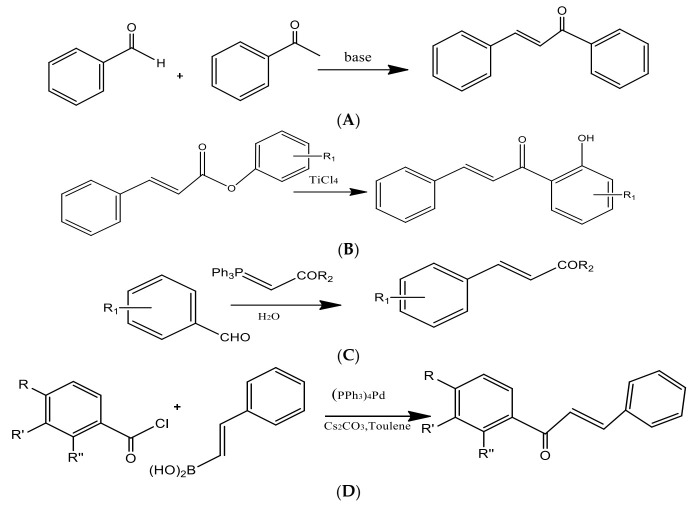
Preparation of chalcones via the (**A**) Claisen–Schmidt condensation, (**B**) Photo-Fries rearrangement, (**C**) Wittig reaction, and (**D**) Suzuki reaction.

Through a green synthesis, different combinations of these ketones and aldehydes have been produced utilizing ethanol as the solvent and a catalytic quantity of HCl to start the Claisen–Schmidt reaction [21] (Figure 4). Dihydrochalcones were created by hydrogenating (using H_2_ gas) the corresponding chalcones in ethanol with palladium on the carbon catalyst. The most popular and often used suitable method for synthesizing chalcones is the conventional Claisen–Schmidt condensation [26] with aqueous alkaline bases, Ba(OH)_2_, LiOH, etc. For the synthesis of chalcones, other well-known methods include the Photo-Fries rearrangement, Wittig reaction, and Suzuki reaction [27]. Using zinc nanoferrite as a catalyst, another proficient and flexible solvent-free synthesis of chalcone derivatives subjected to microwave radiation has been created [28].

## 3. Absorption and Metabolism of Phloretin Using In Vivo Models

As a natural dihydrochalcone flavonoid, phloretin is present in juicy fruits and root peels such as apples, pears, etc. It exhibits many pharmacological properties, such as antidiabetic, antioxidant, anti-inflammatory, antitumor, treating cardiovascular diseases, etc. The pharmacokinetics of phloretin was observed by oral administration as well as an intravenous injection in rat models. Such studies have shown the bioavailability of phloretin to be 8.67% [29]. The absorption and bioavailability of phloretin are poor because of poor water solubility [30], which is dependent upon factors such as physicochemical factors (degree of dissociation, stability in the gastrointestinal tract) and physiological factors (transporters, interaction with serum proteins, pH of the gastrointestinal fluid) [30]. It has been observed that there are few studies related to the characteristics and the mechanism of phloretin absorption [29]. A recent study has shown that the oral administration of phloretin to rats is readily absorbed. It is transported in the plasma and takes nearly 5 h to eliminate 63.2% of the drug, and the process is controlled by the extra-hepatic tissues [31]. After oral administration, it was observed that the plasma concentration–time curve of phloretin showed two peaks. This was attributed to the intestinal–hepatic circulation, double-site absorption, and intestinal efflux [32]. According to an in situ study, the intestinal permeability of phloretin has been observed to decrease with the increase in concentration as the pH of the small intestine changes when phloretin passes through it. In the duodenum, jejunum, and ileum, the pH varies between ~5 and 7 and therefore has various effects on the absorption of phloretin. Previous studies have shown that phloretin is more stable in acidic environments [33]. The study conducted on in vivo rat models ranked the intestinal permeability as colon > duodenum > jejunum > ileum. The absorption of phloretin in the colon region was significantly better than in other regions of the intestine. It was also noted that the clearance of phloretin was 5-fold higher than the mean flow of hepatic plasma [29]. A study has highlighted that the T_1/2_ of phloretin was ~2.82 h, suggesting that it gets eliminated quickly in in vivo models after oral administration. Research is now being focused on enhancing the absorption of phloretin in in vivo models by preparing various dose compositions using nanoemulsion, liposome, and microemulsion [34].

## 4. Anticancer Pharmacological Effects of Phloretin

### 4.1. Apoptotic and Cell Cycle Arrest Mechanisms

**Figure 5:**
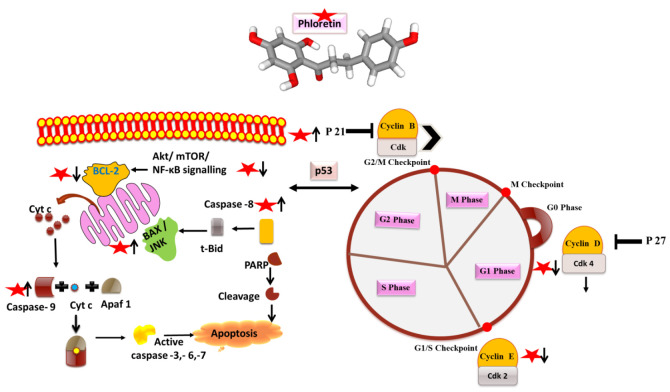
Phloretin (as red star) and its mode of action on apoptosis and cell cycle arrest molecules. Arrows designate up (↑) and downregulation (↓) of the molecules.

Extensive studies on the anticancer activities of phloretin on different cell lines such as prostrate, lung, oral, breast, and liver have been explored and documented [35]. The anticancer activity of phloretin via inducing apoptosis, inhibiting cell growth, and regulating the cell cycle has been reported by many researchers; further studies have also reported the role of phloretin in inducing mitochondrial-mediated apoptosis in cancer cell lines [22,35] (Figure 5). It has been demonstrated that phloretin and phloretin nanoparticles (PhNPs) induce apoptosis in cancer cell lines via the upregulation of BAX, cytochrome c, PARP, caspases 3 and 9, apoptotic activating factor (APAF) and by downregulating the expression of Bcl-2 [15,33]. Mariadoss et al., in their study, demonstrated that positively charged PhNPs resulted in the mitochondrial outer membrane permeability leading to the release of proapoptotic proteins and activation of the mitochondrial-derived caspases, inducing apoptotic cell death [33]. Xu et al., in their study, reported the beneficial effect of phloretin on human gastric cancer in promoting apoptosis via G2/M cell cycle arrest and by diminishing the activity of JNK, thereby resulting in the suppression of cell invasion [11]. In another study, a ruthenium phloretin complex resulted in the apoptosis of breast cancer cells via modulating p53 activity and p53-mediated activation of apoptotic events expedited by the p21, Cyt-C, caspase 9, cleaved caspase 3, Bax signaling and downregulating the Bcl-2 mediated signaling. This study throws light on the beneficial properties of the ruthenium phloretin complex in halting the progression of breast carcinoma, thereby acting as a potential candidate for future cancer chemotherapeutics [15]. Many research groups have highlighted the role of phloretin in the inhibition of the type 2 glucose transporter (GLUT2) in cancer cells and in causing apoptosis, thereby halting the process of metastasis [14,16,36]. Authors have shown that GLUT2 inhibition by phloretin caused a G0/G1 cell cycle arrest in cancer cells, implicating the role of phloretin-induced glucose deprivation, thereby leading to ATP depletion and induction of the mitochondrial death pathway cascade [14]. Different studies on cancer cell lines have shown that phloretin resulted in a cell cycle arrest at different stages, thereby leading to the apoptosis of cancer cells [18,37]. For example, researchers demonstrated that phloretin treatment on HepG2 cells increased the populations of cells in G0/G1 and G2/M stages [37]. Furthermore, in another study by the authors, phloretin treatment to hepatoma cells resulted in a G1 phase arrest, ultimately resulting in a decreased M phase population, suggesting the potential role of phloretin’s aromatic structure in interfering with the DNA double helix, thereby preventing DNA replication, arresting cell cycle, and leading to apoptosis [14]. Studies have thrown light on the role of phloretin in down-regulating Akt and mTOR-mediated signaling, responsible for regulating cyclins, which play a crucial role in controlling the action of enzymes essential for the passage of cells from the G1 to S stages of the cell cycle [13,20,33,38]. In another study, the treatment of colon cancer cells with phloretin resulted in the increased expression of Bax and the subsequent release of Cyt C and DIABLO/SMAC in the cytosol, resulting in the cleavage of caspases-8,9,3,7 and PARP, thereby causing apoptosis of colon cancer cells [13,39]. Studies have also shown that phloretin induces apoptosis in cancer cells via the inhibition of Bcl-2 levels and a decrease in the phosphorylation of c-Jun *N*-terminal kinase (JNK) and p38 MAPK [7,9,20]. In a study conducted by a group of researchers, phloretin resulted in a G0/G1 arrest in human glioblastoma cells and increased the expression of p27, furthering the levels of CDK-2,4,6 and the levels of cyclin D and E that were suppressed, resulting in apoptosis and reduced cell proliferation [40]. Furthermore, many isolated studies have confirmed the role of phloretin in inducing apoptosis and cell cycle arrest, demonstrating its potential benefits in cancer as phytochemical [38,41,42]. In the present scenario, detailed in vivo studies to elucidate the involved pathways and mechanisms, and translate the research from bench to bedside, keeping in mind a potent therapeutic anticancer compound, i.e., phloretin, is needed.

### 4.2. Antiangiogenic and Antimetastatic Action

**Figure 6:**
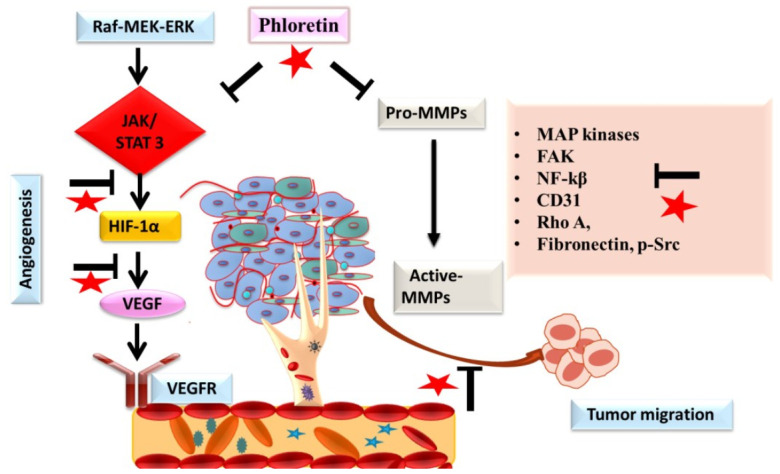
The role of phloretin (as red star) in inhibiting angiogenesis and metastatic spread of cancer cells. Inhibition of the signaling is designated by blockage (⊥) sign.

Metastasis being a multistep process is one of the hallmarks of cancer cells. This process starts with gaining the property of mobility, following next is the detachment of tumor cells and the degradation of the extracellular matrix (ECM) and local invasion, and finally, gaining entry into systemic circulation and extravasation. Different proteins such as MMPs and signaling molecules are involved in the process of metastasis [43]. Angiogenesis under pathological conditions plays an important role in the process of growth, proliferation, and metastasis of cancer cells. Studies targeting angiogenic and metastatic events using different herbal compounds have shown promising results in checking the progression of cancer [43,44]. Different researchers have demonstrated the potential benefits of phloretin in halting the progression of cancer by targeting these angiogenic and metastatic processes [8,20,45] (Figure 6). Numerous studies have reported that phloretin demonstrated protective effects on different cancer cells by significantly decreasing the ability of invasion, migration, activities, and expression of MMP-2, MMP-3 and cathepsin [20,45]. Studies also demonstrated that phloretin affected and reversed the epithelial-mesenchymal transition (EMT), an important step in tumor migration induced by TGF-β1, and also downregulated the levels of Rho A, fibronectin, and p-Src [17]. Studies have reported that phloretin suppressed the expression of VEGF and CD31 in tumor cells, suggesting its role in influencing the process of angiogenesis in cancer. Different studies have shown that phloretin significantly affects the activity of cancer stem cells via targeting different stemness pathways and markers, such as ALDH 1, CD44, CD31, VEGF, Sox-2 etc., thereby diminishing the clonal property of cancer stem cells and reducing angiogenesis, tumor growth, and metastasis [11,16,17]. In another study by Min et al. on lung epithelial cancer cells, a prior incubation with phloretin diminished the migration potential of cancer cells in a concentration-dependent manner via down-regulating the expression of NF-κB and MMP-9 [8]. Similarly, in another study on non-small cell lung cancer cells, phloretin treatment resulted in the suppression of MMP-2 and -9 levels, thereby decreasing the migration potential [46]. In a study conducted by the authors, phloretin and the ruthenium complex halted the process of angiogenesis via down-regulating the EGFR and VEGF signaling in breast carcinoma [13,15]. Studies on different cancer cell lines have demonstrated that phloretin attenuates cell invasion via blocking or decreasing the phosphorylation of MAP kinases, focal adhesion kinases, and Src kinases [16,47]. Phloretin, a polyphenol that is present in apples and in numerous fruits and vegetables, has demonstrated potential benefits in deaccelerating the process of metastasis and angiogenesis in cancer cell lines. However, more in vivo exploratory studies on the potential benefits of phloretin in malignant tissues are needed to validate the long-term applications of phloretin.

### 4.3. Anti-Inflammation and Antioxidative Mechanisms of Phloretin

**Figure 7:**
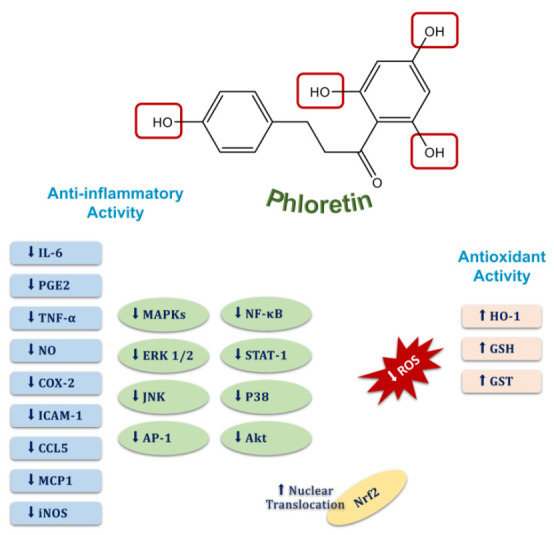
Molecular targets of the anti-inflammatory and antioxidant activities of phloretin.

Numerous preclinical studies have indicated that phloretin has significant anti-inflammatory and antioxidant activity due to its flavonoid structure that has four O-H bonds [20,48] (Figure 7). It is well known that the antioxidant capacities of compounds can be evaluated depending on their bond dissociation enthalpy (BDE) and ionization potentials (IPs) according to the density functional theory (DFT) [19]. Although IPs refer to the energy required to deprive an electron of its quiescent atoms or molecules, BDE is known as a part of the endothermic process and indicates the energy required to break a bond into two isolated atoms or molecules. Therefore, IPs provide information on the efficiency of singlet-oxygen quenching and scavenging of free radicals, and molecules with a low BDE have a better antioxidant capacity and break bonds easily. Considering the BDE and IPs of phloretin, removing an electron from phloretin in water to quench singlet oxygen and scavenge free radicals costs very little energy, allowing it to exhibit an incredible antioxidant capacity [19,49]. It has been shown that phloretin can scavenge peroxynitrite radicals and inhibit lipid peroxidation due to the 2,6-dihydroxyacetone moiety in its chemical structure, and it has been determined that the hydroxyl group at the 20 position of phloretin is an essential pharmacophore for lipid peroxidation and radical scavenging activities [20,50,51]. Moreover, studies to elucidate the molecular mechanisms of phloretin’s antioxidant activity have shown that cellular levels of antioxidant enzymes, such as glutathione (GSH) and heme oxygenase-1 (HO-1), are restored by phloretin due to its significant activity on the redox-regulated transcription factors such as nuclear factor-erythroid related factor-2 (Nrf2) and extracellular signal-regulated kinase (ERK) [52,53]. On the other hand, phloretin also exerts significant anti-inflammatory activity through multiple pathways such as the inhibition of pro-inflammatory signaling pathways and the regulation of inflammation-related transcription factors [54,55]. For example, it has been reported by several studies that phloretin downregulates the activity of inflammation-related factors such as the mitogen-activated protein kinases (MAPKs), the extracellular signal-regulated kinase (ERK) 1/2, the c-Jun N-terminal kinase (JNK), activator protein 1 (AP-1), nuclear factor kappa-light-chain enhancer of activated B cells (NF-κB), signal transducer and activator of transcription 1 (STAT1), and p38 [56,57,58,59,60] (Figure 7). Additionally, the nuclear translocation of Nrf2 has been shown to be increased depending on the activity of phloretin in different experimental models [58,60,61]. Besides enhancing the nuclear translocation of Nrf2, the pretreatment of phloretin in a mouse model of LPS-induced lung injury has also been determined to significantly decrease pro-inflammatory cytokines by reducing p65 and the phosphorylation of the MAP kinases, p38, ERK 1/2, and JNK [54,60]. Furthermore, being associated with the blocking activity of phloretin on the nuclear translocation of NF-κB and the phosphorylation of Akt and MAP kinases, pretreatment of phloretin inhibits the secretion of inflammatory markers such as IL-6, PGE2, TNF-α, and NO and reduces the expression of COX-2, ICAM-1, CCL5, MCP1, and iNOS [57,59,60,62,63,64] (Figure 7). Consequently, phloretin has incredible antioxidant activity due to the O-H bonds in its chemical structure and shows significant anti-inflammatory activity through multiple pathways.

## 5. Synergistic Effects with Other Drugs

The synergistic response between phloretin, palmitoylcarnitine, and ionophore has shown positive results indicating the stimulation of protein kinase C and that it plays a role in the cell cycle [65]. The synergistic effect on breast cancerous cells, such as MCF-7 and MDA435/LCC6, by using phloretin with other flavonoids has been studied on cell cycle proteins [66]. A study has shown that the use of phloretin along with radiotherapy significantly reduces the proliferation of lung tumor tissues. It also enhanced apoptosis and delayed tumor growth [67]. Phloretin and atorvastatin together were observed to produce a powerful synergistic interaction and suppressed colon cancer cell growth. They synergistically induced apoptosis and cell cycle arrest at the G2/M phase [39]. Synergism between phloridzin and phloretin has been observed. Together they act as antineoplastic agents and are being further analyzed to be used as drug candidates [68]. Another study has highlighted the use of phloretin with cisplatin, similarly enhancing apoptosis and cell cycle arrest in the lung cancer A549 cell line. It significantly decreased the levels of Bcl-2 and increased the levels of caspases [69]. The use of phloretin with daunorubicin has been associated with decreasing the levels of HIF-1α and increasing apoptosis in in vitro colon cancer and myeloid leukemia systems [70]. Phloretin with doxorubicin also showed similar effects. The use of phloretin with chaperones (HSP inhibitors) has been associated with enhanced anticancerous activity in myeloid leukemia in murine models [71]. Berbamine with phloretin effectively showed antitumor effects in two SORA-resistant hepatocellular carcinoma cell lines by increasing SHP-1-mediated STAT3 inactivation [72]. Phloretin with chemotherapeutic agents such as tamoxifen and doxorubicin, significantly induced cytoprotective autophagy in the cell lines of breast cancer. It increased the inhibitory effects of cell growth by regulating the mTOR/Akt pathway [41].

## 6. Nano Delivery Studies of Phloretin

Lately, there has been an emphasis on studies related to the nanodelivery of phloretin. A study has shown phloretin loaded with chitosan nanoparticles (NPs) that resulted in pH-dependent mitochondrial-mediated intrinsic apoptosis in human oral cancer cell lines (YD-9, CA9-22). It affected the release of Bax, cytochrome-c, and caspases-3 and -9 [73]. It also influenced the mitochondrial-mediated apoptotic mechanism by stimulating oxidative stress [33]. The development of a fast-dissolving nanofiber (NF) of phloretin has shown >90% drug delivery efficiency in the treatment of oral cancers. Higher levels of apoptosis were observed in cancer cell lines treated with a PVA/TPGS/phloretin NF group compared to phloretin alone [74]. Phloretin conjugated with AuNPs showed enhanced antineoplastic activity in the human cervical cancer HeLa cell line [68]. Encapsulation of phloretin into the PLGA-NH2 NPs has been used for tumor-targeted delivery in Hep-2 cells (human laryngeal carcinoma) [75]. In another study, chitosan-coated NPs containing phloretin (CS–PLGA/Phl) were tested in mouse melanoma cells (B16F10) and in vivo models [76]. Nanostructured lipid carriers for the encapsulation of phloretin have been proven to be beneficial as they enhance the solubility and efficiency of the drug [77]. Phloretin in the form of an NLC showed a sustained pattern of drug release and absorption in in vitro [77]. Recently, an innovative hydrogel containing polymeric nanocapsules loaded with phloretin has been developed, which enhanced skin penetration and adhesion, and was therefore used to treat skin cancer-related issues without showing any side effects [76]. Recently, cross-linked poly(cyclotriphosphazene-co-phloretin) microspheres have been developed and are being tested for their application for controlled drug delivery in the treatment of cancer [78].

## 7. Phloretin as a Membrane-Disrupting Pan-Assay Interference Compound (PAIN)

Recently, phloretin was identified as a Pan-Assay Interference compound (PAIN) based on its ability to interfere with bioassays through many different mechanisms [79]. Indeed, it was shown that phloretin could absorb into lipid surfaces and modify the dipole potential of lipid layers, particularly in cholesterol-rich domains, leading to disruption of cell membrane homeostasis and alterations in membrane permeability for a wide range of compounds [80,81,82,83]. Therefore, it is possible that phloretin-induced changes in the transmembrane protein conformation and function may somewhat also impact the results of different cell-based anticancer assays. Interestingly, the introduction of a C-glucoside moiety into a phloretin molecule resulted in a derivative, nothofagin, with no ability to alter the membrane dipole potential [83]. Although nothofagin has been characterized by its anti-inflammatory [84,85], diuretic [86], and nephroprotective properties [87], no anticancer activities have been published for this compound so far. Another possibility to overcome the cell membrane disruptive effects of phloretin is to incorporate this dihydrochalcone into nanocarrier-based drug delivery systems. As described in Section 6, this strategy has successfully generated a number of promising results against different cancer types.

## 8. Safety Studies with Phloretin

Phloretin safety concerns cannot currently be stated due to a lack of sufficient data. In fact, only a few experimental studies have been performed with structural derivatives of phloretin (and not the parent compound itself) to confirm this presumption. Both the acute oral toxicity test in Kunming mice and the 30-day feeding subchronic oral toxicity test on Sprague Dawley rats showed no toxicity of aminoethyl-phloretin, a water-soluble derivative of phloretin. In the first experiment, this derivative was injected intragastrically to mice at doses of 2, 4, and 8 g/kg body weight; in the subacute toxicity test, aminoethyl-phloretin was administered by intragastric injection to rats at concentrations of 10, 50, and 100 mg/100 g body weight [88]. No adverse reactions were also observed when a semisynthetic derivative of phloretin, phloretin 3′,3-disulfonate (0.1%), was topically applied to human skin [89]. Another study has revealed that the LD_50_ dose was 400 mg/kg of the ruthenium-phloretin complex [36]. Another study has shown that phloretin showed comparatively less cytotoxicity on gastric GES-1 cells, with an IC_50_ of 120 μM [90]. Similarly, phloretin suppressed the proliferation rate of the human SCC-1 oral cancer cells and showed an IC50 of 12.5 µM [9]. Without any doubt, further studies focusing on the safety issues of the parent phloretin are highly required before any clinical trials can be introduced to assess the anticancer potential of this bioactive dihydrochalcone in human beings. Table 1 represents a summary of various anticancer studies of phloretin.

## 9. Conclusions and Future Perspectives

**Figure 8:**
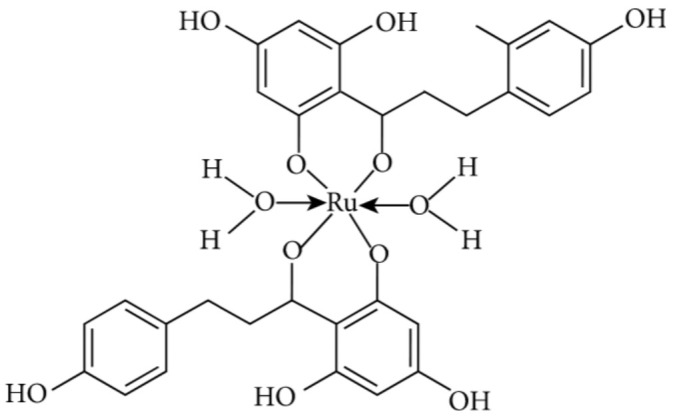
Ruthenium metal complexation of phloretin [36].

In this review article, the current knowledge about diverse types of anticancer effects of a minor flavonoid, phloretin, was compiled, demonstrating antioxidant, anti-inflammatory, antiproliferative, apoptotic, antimetastatic, and antiangiogenic activities of this dihydrochalcone. The multitargeted anticarcinogenic abilities of phloretin clearly show that besides a huge number of common flavonoids, minor flavonoids (including chalcones, aurones, dihydrochalcones, and dihydroflavonols) deserve much more attention, harboring promising therapeutic potential. Safety aspects and caution regarding the PAINS issue strongly compromise the potential of phloretin and its structurally close derivatives to becoming drug candidates for clinical trials. Moreover, by analyzing the structure–activity relationships and relying on the modern synthetic metal complexation (Figure 8), it could be possible in the future to attain a phloretin-based drug candidate for fighting against different cancer types. Further investigations into the *C*-glucosylated derivative of phloretin, nothofagin, are also highly encouraged; differently from the parent compound, this derivative has no modifying effects on the cell membrane dipole potential. Ultimately, considering the ever-increasing incidence of malignant disorders all over the world, the importance of all these studies cannot be emphasized enough.

## Figures and Tables

**Table 1:** Representation of various anticancer studies of phloretin. Arrows designate up (↑) and downregulation (↓) of the molecules.

S. No	Types of Cancer	Cell Culture Model		Mechanisms	Dose/Concentration Used	Ref.
1	Human breast tumor cells	H-Ras MCF10A	Apoptosis induction	↑p53, ↑Bax, and ↑ cleavage of poly (ADP)-ribose polymerase	100 μM	[91]
2	Human triple-negative breast cancer	MDA-MB-231 and BALB/c nude mice	Anti-proliferative and inhibited migration	↓paxillin/FAK, ↓Src, ↓GLUTs, ↑p53, ↑p21, ↑E-cadherin	50–150 μM	[16]
3	Human cervical cancer	SiHa, and nude mice (in vivo)	Anti-metastasis and anti-angiogenesis	↓invasion, ↓MMP-2 ↓MMP-3, ↓cathepsin S	60 μM, 100 μM (in vitro cell lines); 10 or 20 mg/kg (in vivo model)	[17]
4	Human colon cancer	HT-29-Luc cells	Apoptosis induction	↓BCL2, TRAIL	-	[12]
5	Human colorectal cancer	COLO 205 and HT-29 and in vivo BALB/c nude mice	Inhibit cell invasiveness	↓Glucose transporters, ↑p53	12.5, 25, 50, 100, and 200 μM	[36]
6	Human esophageal cancer	EC-109 cells	Apoptosis induction	↓B-cell lymphoma 2 (bcl-2), ↑p53, ↑apoptotic protease activating factor-1	60, 70, 80, 90, and 100 µg/mL	[7]
7	Human Gastric Cancer	AGS	Apoptosis induction and cell cycle arrest	↑G2/M phase arrest	-	[11]
8	Human glioblastoma cells	U87 and U251	Apoptosis induction and cell cycle arrest	↑p27, ↓cdk2, ↓cdk4, ↓cdk6, ↓cyclin D and ↓cyclin E, ↓PI3K/AKT/mTOR	200 μM	[38]
9	Human leukemia cells	HL60	Apoptosis induction	↓protein kinase C	0.1–0.2 mM	[92]
10	Hepatocellular carcinoma	HepG2, SK-Hep1, Sor resistant HepG2^SR^, Huh7^SR^ xenografts	anti-proliferative anti- angiogenesis,	↓STAT3, ↓AKT/mTOR/JAK2/VEGFR2	50 μM	[93]
11	Human liver cancer	HepG2 cells as well in vivo mice models	Apoptosis induction	↓Akt and Bcl-2, GLUT2	200 μM	[14]
12	Non-small cell lung cancer (NSCLC) cells	A549	Apoptosis induction	↑BAX, ↑cleaved caspase-3 and -9, ↑ PARP, ↓Bcl-2	20 mg/kg (administered to in vivo models)	[8]
13	Human oral cancer	SCC-1	Cell cycle arrest	↑G0/G1 cell cycle arrest, ↓cyclin D1, ↓CDK4 and ↓CDK6, ↑ROS	-	[9]
14	Human Prostate Cancer	LNCaP, CWR22Rv1, PC-3, and DU145	Apoptosis induction	↓Sp3/4, VEGF, Survivin	20, 50, and 100 μM	[94]
15	Mouse melanoma	4A5 cells	Apoptosis induction	↑Bax, ↑caspases activation	0.1–0.2 mM	[92]

## Data Availability

Not applicable.
